# Molecular identification of *Kudoa thyrsites* parasite isolated from Atlantic mackerel (*Scomber scombrus*) landed in Ireland

**DOI:** 10.1007/s00436-026-08729-8

**Published:** 2026-07-15

**Authors:** Viktória Királyová, Deirdre Brophy, Lucilla Giulietti, Anita Talbot, Michael Gallagher, Andrew Campbell, Katie O’Dwyer

**Affiliations:** 1 Marine and Freshwater Research Centre, Atlantic Technological University (ATU), Galway, Ireland; 2 Institute of Marine Research (IMR), Bergen, Norway; 3 Bord Iascaigh Mhara (BIM), Killybegs, Ireland; 4 Foras na Mara – Marine Institute, Oranmore, Ireland

**Keywords:** parasitology, myxosporean, fish biology, seafood quality

## Abstract

Post-mortem myoliquefaction, commonly referred to as ‘jelly flesh’, is associated with infection by myxozoan parasites of the genus *Kudoa*, particularly *Kudoa thyrsites;* the effects of infections can render fish fillets unusable in food preparation. Although the parasite has been documented in Atlantic mackerel (*Scomber scombrus*) across parts of the Northeast Atlantic, its presence in mackerel landed in Ireland has not been previously confirmed. In this study, mackerel obtained from commercial landings were screened for signs of post-mortem myoliquefaction. Three individuals displaying characteristics of softening of the musculature were examined using light microscopy and molecular analysis. Microscopic examination revealed numerous spores consistent with *Kudoa-*like morphology. Sequencing of the amplified region confirmed the presence of *K. thyrsites* in all three samples. The sequences showed a 100% match with *K*. *thyrsites* isolates previously reported from Atlantic mackerel and other host species in the Northeast Atlantic. Moreover, phylogenetic analysis placed the Irish isolate within the expected Atlantic sub-clade. As molecular testing was limited to visibly affected fish, the prevalence of *K. thyrsites* infection in the mackerel population could not be estimated, highlighting the need for continued monitoring of the parasite in Irish waters to better understand its distribution and potential economic implications for the Northeast Atlantic fishery.

## Introduction

Post-mortem myoliquefaction, commonly called ‘jelly flesh’ or ‘soft flesh’, refers to the disintegration of fish muscle tissue after death and is associated with parasitic infection (Moran et al. 1999; Levsen 2015). Several myxozoan parasite species of the genus *Kudoa* are known to cause this condition in commercially important fish species, including Atlantic salmon (*Salmo salar*), Atlantic mackerel (*Scomber scombrus*), olive flounder (*Paralichthys olivaceus*), broadbill swordfish (*Xiphias gladius*), and yellowfin tuna (*Thunnus albacares*) (Moran et al. 1999; Levsen 2015; Yokoyama et al. 2017; Bolin et al. 2021). Post-mortem myoliquefaction can render fish fillets unmarketable and decrease consumer confidence, leading to substantial economic losses in fisheries and aquaculture industries worldwide (Moran et al. 1999; Kristmundsson and Freeman 2014).

Atlantic mackerel (*Scomber scombrus*) has a wide distribution in the Northeast Atlantic, spanning from the southern regions of the Iberian Peninsula to the coast of Norway and Iceland (Trenkel et al. 2014). The species’ high value, estimated at over €615 million at first sale in the European Union alone in 2025, makes it an attractive target for commercial fisheries, exploited by several European countries across its distribution (EUMOFA 2025). Mackerel is exported worldwide as fresh or frozen whole fish, while filleting is increasingly used as a way to add value. Since the bulk of the mackerel is processed frozen, ‘jelly flesh’ affected individuals can go undetected until they reach the final consumer. The condition was first identified as a potential issue for the Atlantic mackerel fishing and processing industry in the early 2000s (Levsen 2015). *Kudoa thyrsites* has been unveiled as the causative agent of the condition in mackerel caught in the Norwegian Sea, North Sea, and the vicinity of the Faroe Islands (Giulietti et al. 2022a; Soares et al. 2023; Højgaard et al. 2022).

Like many other myxosporeans, *K. thyrsites* has a complex life cycle involving a fish host and potentially an annelid host (Eszterbauer et al. 2015). In myxosporean parasites, the infective actinospores are released from an annelid into the water column. Once a fish is infected, the sporoplasm develops within the muscle tissue, where myxospores are formed intracellularly within pseudocysts inside muscle fibres (Feist et al. 2015). It is important to note that the life cycle of *K. thyrsites* is not yet fully understood, and the pathways of infection and transmission remain unknown (Eszterbauer et al. 2015). The infection does not cause mortality of the fish host, and ‘jelly flesh’ only manifests 12-48 h after death due to the release of the proteolytic enzyme cathepsin L by the parasite (Funk et al. 2008). However, not all fish individuals infected by *K. thyrsites* will develop the condition, and the degree of muscular degradation seems to be influenced by parasite load in skeletal muscle (Giulietti et al. 2022a).

Developing a robust comprehension of *K. thyrsites* infection and ‘jelly flesh’ occurrence across the distribution of Atlantic mackerel is essential to assess its impact on the pelagic fishing industry in the wider Northeast Atlantic region. The waters to the west of Ireland are important fishing grounds for Atlantic mackerel and an area where fish aggregate for spawning and feeding (Brunel et al. 2018). However, the prevalence and effect of *K. thyrsites* in this area are not known. Here, we aim to identify the causative agent of post-mortem myoliquefaction in Atlantic mackerel landed in fishing ports in Ireland and to compare this to previous findings using established molecular techniques that allow for accurate detection (Giulietti et al. 2022a).

## Methods

**Fig. 1:**
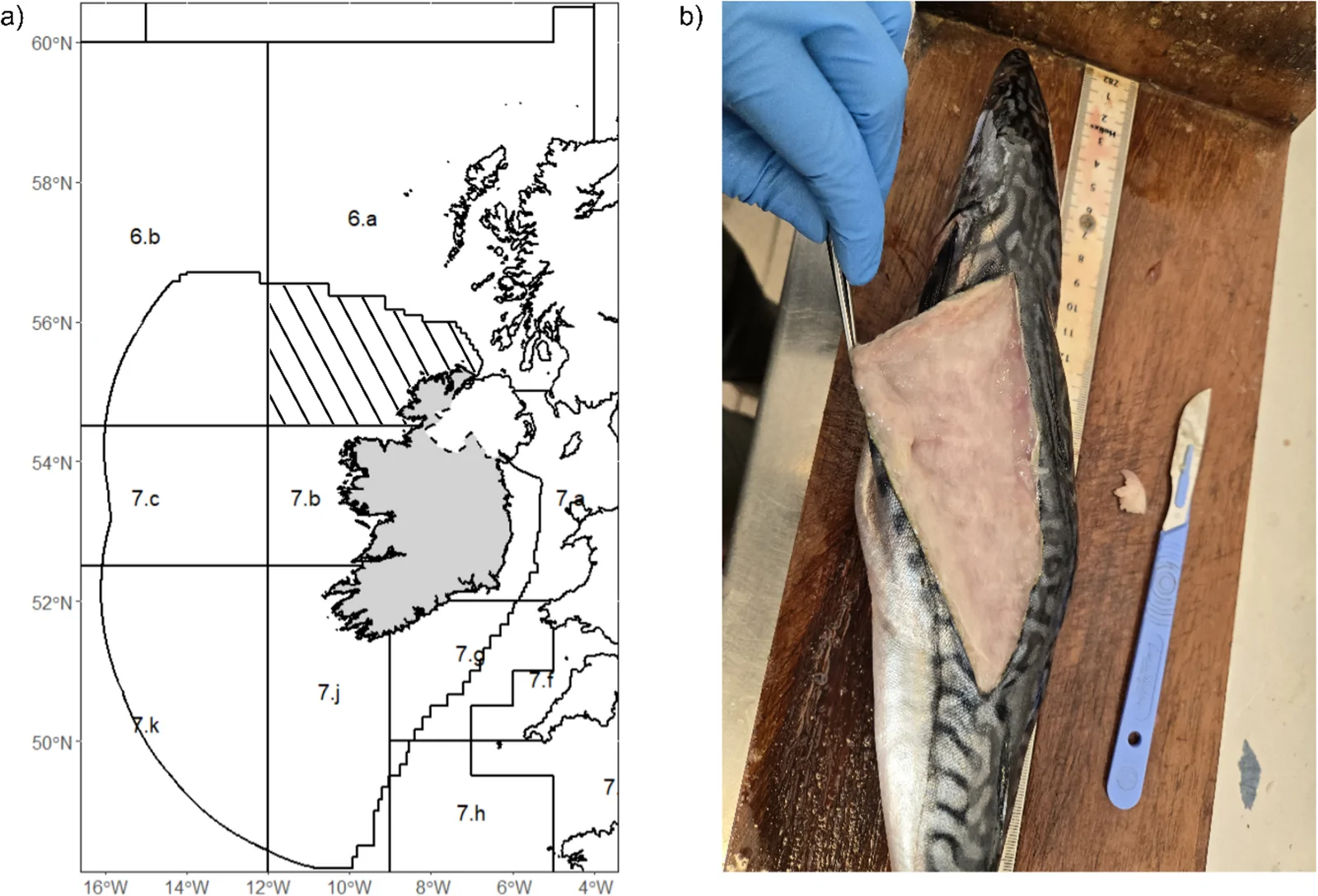
**a)** A map of the Irish exclusive economic zone (EEZ), showing the ICES division 6a. The individuals included in this study were caught within the dashed area of the EEZ and ICES area 6a. **b)** Dorsal incision of Atlantic mackerel displaying ‘jelly flesh’

In total, 1,935 Atlantic mackerel were screened from commercial landings for this study. The fish were caught in the International Council for the Exploration of the Sea (ICES) fisheries management Division 6a in the Northeast Atlantic (Fig. 1). Forty-eight hours following landing, mackerel were inspected, and muscle texture was examined for abnormal softness by pressing along the fish from the dorsal fin to the tail. Fish showing light to clear signs of ‘jelly flesh’ were subjected to further visual inspection of the muscle appearance, to establish whether or not the basic segmental myomere structure was intact (Giulietti et al. 2022b). Based on the muscle examination, three specimens of commercially sized Atlantic mackerel (295 g, 370 g, 597 g) were suspected of displaying ‘jelly flesh’. Two sets of muscle tissue samples from the three individuals were dissected from the dorsal area, which typically shows a high density of *K. thyrsites* in infected mackerel (Giulietti et al. 2022b). The samples intended for light microscopy were stored frozen (-20 °C), and the samples used for genetic analyses were preserved in molecular-grade ethanol (≥ 99.8%).

For microscopic screening, muscle tissue samples were minced using a scalpel and Giemsa stained on a glass slide (Levsen 2015). Slide preparations were initially examined by light microscopy using Olympus BX41 (Tokyo, Japan) at 400x magnification for the presence of *Kudoa*-like myxospores (Lom and Arthur 1989). Preliminary identification of spores was based on their overall appearance and morphology (Lom and Arthur 1989). Subsequent images were taken at 1000x magnification by an Olympus SC50 (Tokyo, Japan) camera.

DNA was extracted from 13 mg of ethanol-preserved muscle tissue from each affected fish using the DNeasy Blood and Tissue Kit (Qiagen, Hilden, Germany) following the manufacturer’s instructions. DNA quality was checked on NanoDrop One (ThermoFisher Scientific, USA) and quantified by Qubit 3.0 Fluorometer (ThermoFisher Scientific, USA). The partial small subunit SSU rDNA (18S) sequence was amplified by polymerase chain reaction (PCR) using the primer pair K.thyr18Sfor_3 (5’-GGT CAT ATG CTC GTC TCA AAG-3’) and K.thyr18Srev_3 (5’-TCG GTC AAG ACA ATT TAA CCG-3’) following standard cycling conditions (Levsen 2015). The reaction was carried out in a 50 µl volume containing 25 µL of Dream Taq (ThermoFisher Scientific, USA), 2 µL of each 100 nM primer, 100 ng DNA template and nuclease-free water. PCR products were purified using the QIAquick PCR Purification Kit (Qiagen, Hilden, Germany). The purified products were diluted to 10 ng/µL and prepared for sequencing by adding 10 µM of amplification primers. Sequencing was performed in both directions for increased accuracy by Eurofins (Cologne, Germany).

Sequences were assembled and trimmed in ChromasPro 2.1.5 (Technelysium Pty Ltd., Australia). The identity of the isolate and comparison to previously deposited *Kudoa* species were verified in GenBank using BLAST searches (BLAST: Basic Local Alignment Search Tool). Based on BLAST scores, geographical locations and host species, twenty *K. thyrsites* SSU rDNA nucleotide sequences were chosen for phylogenetic analyses. Outgroup taxa were *K. septempunctata* and *K. igami*. Sequence alignment and construction of the phylogenetic tree were carried out in MEGA 12 (Kumar et al. 2024). The phylogeny was inferred using Maximum Likelihood (ML) with nodal support inferred based on 1000 bootstrap replicates.

### Results & discussion

**Fig. 2:**
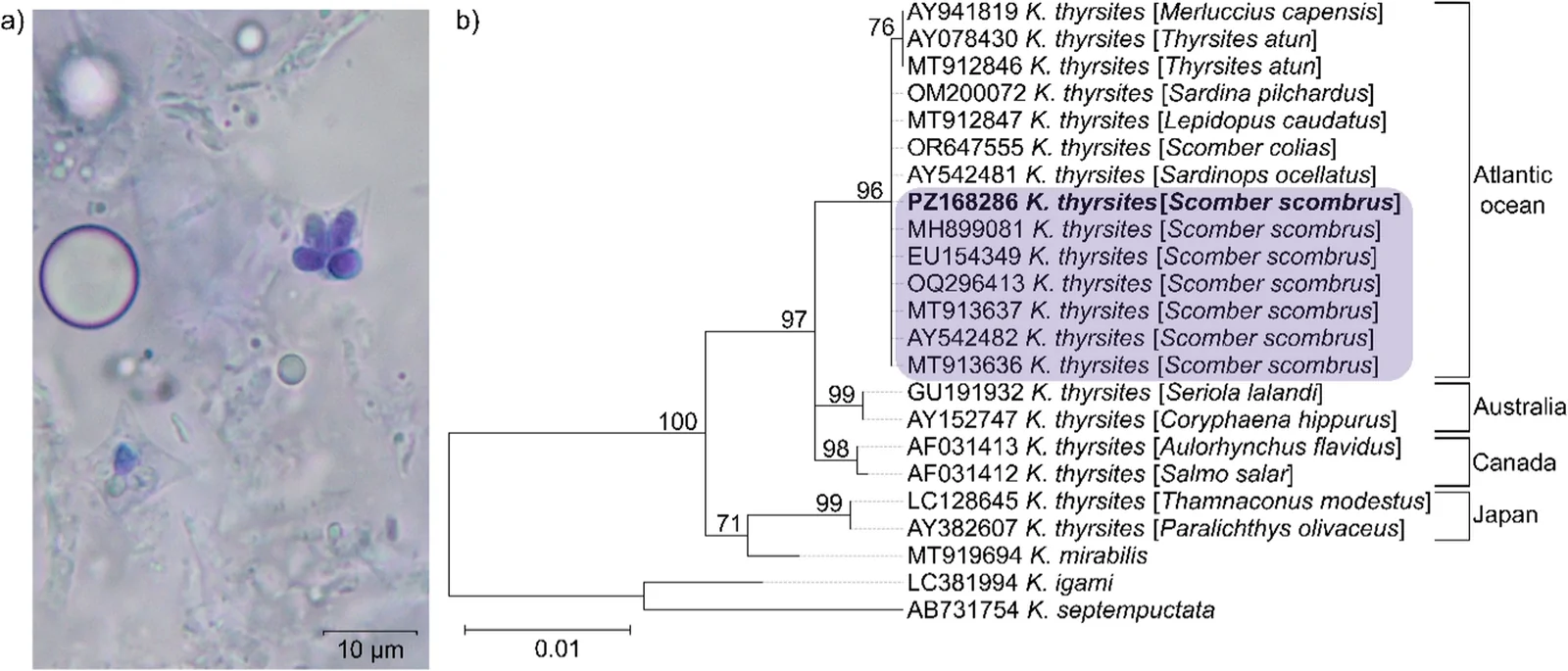
**a)** *Kudoa* myxospores, 1000x magnification. **b)** Phylogenetic tree for *Kudoa* species constructed based on comparison of the SSU rDNA. Isolates of *K. thyrsites* from Atlantic mackerel are highlighted in purple, including our isolate in bold. The percentage of replicate trees in which the associated taxa cluster together (1000 replicates) is shown above the branches

During microscopy, numerous myxosporean spores were observed that exhibited features typical of *Kudoa-*like parasites (Fig. 2a). The spores consisted of four pyriform polar-like capsules, with one capsule considerably larger than the others (Lom and Arthur 1989; Giulietti et al. 2020). No inflammatory reactions were observed in the musculature of the specimens. The presence of *Kudoa thyrsites* in Atlantic mackerel landed in Ireland was confirmed by sequence analysis of the SSU rDNA (18 S) from all three ‘jelly flesh’ specimens collected. The Irish isolate was submitted to GenBank with the following accession number (PZ168286). The obtained amplicon of 1187 bp matched among the three samples and had 100% identity with *K. thyrsites* from mackerel in the wider Northeast Atlantic region (Fig. 2b). Based on the phylogenetic analysis, our isolates clustered within the Atlantic sub-clade and showed 100% identity with *K. thyrsites* isolates from mackerel in the North and Norwegian Seas (GenBank accession nos. EU154349, OQ296413, AY542482, MH899081, MT913637, MT913636), as well as from other fish species from the waters of Spain, Morocco, South Africa and the Mediterranean Sea (GenBank accession nos. OM200072, OR647555, AY542481, MT912847). The sequences showed close identity (1186/1187 bp) to two isolates from South Africa and one from Namibia (GenBank accession nos. AY941819, AY078430 and MT912846), clustering within the Atlantic sub-clade. Additionally, they differed from isolates from Australia (1183/1187 bp) and Canada (1182/1187 bp), each forming a separate sub-clade, while the isolates from Japan (1175/1187 bp) formed another distinct sub-clade in a sister relationship with *K. mirabilis* (Fig. 2b). The tree topology based on our phylogenetic analysis is in agreement with previous studies (Whipps and Kent 2006; Giulietti et al. 2020).

Although *K. thyrsites* has been detected in Atlantic mackerel across several parts of the Northeast Atlantic, information on the parasite’s temporal dynamics is available only for Norwegian waters (Giulietti et al. 2022a). The long-term study on *K. thyrsites* in Atlantic mackerel from the Norwegian fishery provides important context for understanding the epidemiology and temporal patterns of the parasite in the Northeast Atlantic. Their monitoring programme demonstrated that although baseline occurrence of ‘jelly flesh’ in mackerel remained relatively stable at approximately 0.6% between 2009 and 2018, a notable increase was observed in subsequent years, with prevalence peaking at 3.66% in 2020 (Giulietti et al. 2022a). These fluctuations indicate that infection pressure, albeit low in the region, is not static and may respond to broader ecological changes and anthropogenic pressure on mackerel stock (e.g. changes in water circulation, host migration patterns, alternate host occurrence, benthic habitat, host density and fishing pressure) and requires ongoing investigation.

Atlantic mackerel start their annual spawning migration around the turn of the year when they depart overwintering grounds in the Northern North Sea and migrate to southern waters to spawn, with the majority of spawning taking place in spring and early summer in the Bay of Biscay and waters to the west of Ireland (Brunel et al. 2018). Following spawning, they return to feeding grounds in the Norwegian Sea. Throughout their migration and spawning period, mackerel from across the Northeast Atlantic mix extensively, resulting in a genetically homogenous stock with no discrete spawning components (Manuzzi et al. 2025). Tagging and recapture data further show that migration patterns are size dependent, with larger and older individuals typically travelling greater distances, and tending to remain at the leading edge of the migration (Ono et al. 2022). These extensive movements and repeated mixing suggest that parasite infections are unlikely to be spatially restricted. However, because larger fish also tend to carry higher parasite burdens and aggregate within certain parts of the migration route, localised increases in prevalence may still occur (Giulietti et al. 2022a; Ono et al. 2022). In addition, in areas with greater availability of the unidentified annelid host and host-parasite encounter rates, fish may be more prone to acquiring new infections. Due to extensive movement of mackerel into the area west of Ireland during the spawning season, it is important to establish how the prevalence of the parasite changes spatially and temporally within this area. Combined with the limited understanding of the parasite’s complex life cycle, these uncertainties make it difficult to predict how environmental change or anthropogenic pressure might alter infection rates and ‘jelly flesh’ occurrence dynamics.

In conclusion, this study confirms *K. thyrsites* as the causative agent of ‘jelly flesh’ in Atlantic mackerel landed in Ireland. Through future research and systematic sampling, we aim to determine the scale of parasite prevalence. Given that even a small number of affected individuals may have a negative impact on product quality and market perception (Giulietti et al. 2022a; FSAI 2025), we believe sustained monitoring in Irish waters is essential for assessing potential economic impacts of ‘jelly flesh’ on the Northeast Atlantic mackerel fishery.

## Data Availability

Sequence data were deposited into the GenBank database under accession number PZ168286 and are available at the following URL: https://www.ncbi.nlm.nih.gov/nuccore/PZ168286.
